# The influence of zinc-methionine bioplex supplementation to pregnant and lactating sheep on selected wool parameters

**DOI:** 10.5194/aab-62-99-2019

**Published:** 2019-03-21

**Authors:** Anna Wyrostek, Stefania Kinal, Bożena Patkowska-Sokoła, Robert Bodkowski, Paulina Cholewińska, Katarzyna Czyż

**Affiliations:** 1 Institute of Animal Breeding, Wrocław University of Environmental and Life Sciences, Wrocław, 51-630, Poland; 2 Department of Animal Nutrition and Feed Management, Wrocław University of Environmental and Life Sciences, Wrocław, 51-630, Poland

## Abstract

The most important nutrients affecting wool production include sulfur amino acids, such as methionine, and minerals, such as zinc, and their deficiency may cause wool production decrease. The aim of this study was to evaluate an effect of zinc and methionine chelate addition on the qualitative characteristics of Merino ewes wool and zinc content in wool fibers and on their surface. Histological evaluation of fibers was also performed. The study included 22 Polish Merino ewes divided into two groups: control and experimental (0.4 g chelates daily per head). The experiment lasted for 4 months (3.5 months of pregnancy and 2 weeks of early lactation). The length, thickness and breaking force of wool fibers were higher in the experimental group compared to the control group. Higher content of zinc in wool of sheep from the experimental group was also demonstrated. The histological structure of fibers from both groups did not differ, as the content of zinc on their surface. It can be concluded on the basis of the obtained results that the addition of chelates favorably influenced the thickness compensation of wool fibers and amount of wool obtained from sheep during pregnancy and early lactation, as well as mechanical features of wool.

## 1 Introduction

Wool is a natural raw material obtained mainly from sheep, but also from alpacas, llamas, some breeds of goats and rabbits. The production and quality of wool are affected by numerous factors that include breed, sheep genetics, age of the animal, part of animal body the wool is collected from, color of wool fibers, intervals between successive shears, and some environmental and management factors (Reis and Sahlu, 1994; Sahoo and Soren, 2011; Holman and Malau-Aduli, 2012; Khan et al., 2012; Ragaišienė et al., 2016). One of the main factors affecting the quantity and quality of wool produced is proper nutrition of the animals. The most important nutrients that affect its production include sulfur amino acids (cystine, cysteine and methionine) and lysine. Cystine and cysteine are the most important elements in the growth of wool, but methionine can be transformed into them through transsulfuration and transamination pathways. These processes involve the contribution of 3/4 of methionine supplied to the organism. Methionine is considered an indispensable amino acid for animals, and it is known that it cannot be synthesized to a degree that would be sufficient to maintain the proper growth and development of animals (Reis, 1989; Reis and Sahlu, 1994; Sahoo and Soren, 2011; Khan et al., 2012; Bin et al., 2017). In addition to its role in wool production, methionine is also involved in oxidative stress reduction; its deficiency is linked to hepatic pathology, intestinal epithelium growth suppression and growth performance disorders (Bauchart-Thevret et al., 2009; Bin et al., 2017).

Moreover, some minerals, such as zinc, also affect the production of wool. This effect can be due to reduced feed intake and altered function of the rumen and thus nutrient supply or metabolic processes disturbances (Szigeti et al., 2016). The deficiency of zinc in the feed ration is manifested by an inhibition of wool growth, loss of wool and an increased wool fiber fragility (Sahoo and Soren, 2011; Khan et al., 2012). Except its role in wool production, zinc affects a wide variety of functions, including gene expression, DNA and protein synthesis, cell signaling, cell division and growth of the animal, development and function of the immune system, synthesis of structural proteins like collagen and keratin, bone and tissue development, reproduction and the organism's defense against oxidative stress (Sun et al., 2011; Sloup et al., 2017). Deficiency or a marginal zinc status may impair one or more of these processes; therefore, this element is widely supplemented in animal diets either in the form of inorganic salts (e.g., sulfates, chlorides or oxides) or in organic forms. The advantage of inorganic forms is their low cost; however, it is generally believed that this form is less bioavailable compared to the organic one. Many authors compared in their research the bioavailability of inorganic and organic forms of zinc in the nutrition of animals, especially ruminants (Spears, 1989; Rojas et al., 1995; Cao et al., 2000; Ryan et al., 2002; Pal et al., 2010; Hassan et al., 2011; Jafarpour et al., 2015; Page et al., 2017). These studies demonstrated better bioavailability of organic forms (bioplexes), so-called chelates, i.e., compounds of metal ions and the ligand (protein or amino acid). They do not demonstrate any antagonisms and interactions that can occur between inorganic salts and other components like hydroxides, phosphates, carbonates and oxygen, which ensures highest bioavailability of organic forms (Hosnedlova et al., 2007; Kinal and Slupczynska, 2011; Sahoo et al., 2014). The chelates also retain stability at varying pH levels in the digestive tract (Hassan et al., 2011).

Pregnancy, and especially its second part and early lactation, exhibits a negative impact on wool production in the ewes (Corbett, 1979; Masters et al., 1993; Reis and Sahlu, 1994; Khan et al., 2012). It was demonstrated that improper nutrition of sheep during pregnancy negatively affects the development of secondary hair follicles in their lambs, resulting in permanent reduction in adult wool production (Szigeti et al., 2016). Also, Thompson et al. (2011) showed that the nutrition and condition of ewes during pregnancy affects the production and diameter of the wool fibers of their lambs.

Many studies indicate that wool is a good indicator of micro-element supply to the organism and its analysis can be used as an alternative test to the analysis of minerals in other tissues, such as blood (Hawkins and Ragnarsdóttir, 2009; Patkowska-Sokoła et al., 2009).

**Table 1:** The share of particular components in basic diet for pregnant and lactating ewes (%)${}^{*}$.

Diet	Pregnant	Lactating
component	ewes	ewes
Maize silage	34.4 %	35.8 %
Meadow hay	27.4 %	25.9 %
Rye straw	27.4 %	21.4 %
Barley grain	13.8 %	17.4 %

${}^{*}$ Content per kilogram of dry matter.

The aim of this study was to evaluate an effect of chelates (Zn + methionine, denoted ZnMet), in the form of a Zinpro100 preparation, on wool parameters of Polish Merino ewes during pregnancy and early lactation, including physicomechanical characteristics of wool (length, diameter, breaking force, elongation, tensile strength), content of zinc in wool, composition of elemental ions on the surface of wool fibers and histological structure of the fibers.

## 2 Material and methods

The study involved 22 Polish Merino ewes at the age of about 3 years that were kept in the sheepfold belonging to the Agrominor company in Mokrzeszów (Lower Silesia province, Poland). The manner in which the experiment was conducted ensured animals welfare, and did not cause any unnecessary pain, stress or discomfort according to the act of 15 January 2015 on the protection of animals used for scientific and educational purposes (Journal of Laws, 2015). They were randomly selected and divided into two equal groups: control, without the supplementation, and experimental, receiving the Zinpro100 supplement with an amount of 0.4 g per head per day. The sheep were maintained in the sheepfold building in separated group pens on a deep straw litter. The experiment lasted for a total of 4 months and included 3.5 months of pregnancy and 2 weeks of lactation. All sheep, both during pregnancy and lactation, were fed in the same manner (Table 1). The content of zinc in the control dose during pregnancy was 49.36 mg kg${}^{-1}$ DM (dry matter), and during lactation it was 62.12 mg kg${}^{-1}$ DM; in the case of sheep from the experimental group it was 89.36 and 102.12 mg kg${}^{-1}$ DM, respectively (Table 2).

In order to unify the wool regrowth in all sheep, a 10cm×10 cm patch was cut on the left side at the height of the last rib of each sheep on day 0. After a 4-month period of the experiment, the wool was cut again at this point, and it was used for the measurements of the selected physicochemical parameters of the fibers, determination of zinc content in wool and elemental composition of wool fibers, as well as characterization of the differences in histological structure of wool fibers.

Prior to measurements, the fibers were washed with a mild detergent in order to remove suint from their surface. All measurements were made in the middle part of the fibers.

**Table 2:** Zinc content in feeding dose for pregnant and lactating ewes (mg kg${}^{-1}$ DM).

Group	Pregnant	Lactating
	ewes	ewes
Control	49.36	62.12
Experimental	89.36	102.12

The diameter of wool fibers was measured using an MP3 lanameter with a magnification of 500×, according to the standards approved for wool thickness analysis (European Union, 2016). Fiber length was determined without fiber stretching using a ruler and 10× magnifying glass; for this purpose, the fibers were mounted on slides and immersed in paraffin oil. Fiber diameter and length measurements were made for 300 fibers from each animal.

Breaking force (neutons, N) and elongation (percent, %) were determined for 20 fibers from each animal using a Matest (Poland) tensile testing machine and software. Tensile strength was calculated according to the following formula:

1 $$R_{S}=\frac{F}{A}\;\left(N\;{\mathrm{mm}}^{-2}\right),$$

where F is breaking force (newtons, N) and A is hair fiber cross section (mm${}^{2}$).

The measurements were made at the Skin and Hair Coat Laboratory of the University of Life Sciences in Wrocław, Poland.

**Table 3:** Selected physicomechanical parameters of wool samples.

Parameter	Group	
	Control	Experimental
Length (cm)	$2.58^{A}\pm 0.50$	$3.31^{B}\pm 0.88$
Thickness (µm)	$22.56^{A}\pm 0.35$	$24.39^{B}\pm 0.80$
Breaking force (N)	$0.10^{A}\pm 0.01$	$0.12^{B}\pm 0.01$
Elongation (%)	$40.29^{a}\pm 1.56$	$38.82^{b}\pm 1.74$
Tensile strength (N mm${}^{-2}$)	255.78±27.75	252.19±39.02

${}^{A,B}$ – differences significant at P≤0.01; ${}^{a,b}$ – differences significant at P≤0.05.

Determination of zinc content in wool was made by atomic spectrometry at the laboratory of the Department of Nutrition and Feed Management of the Wrocław University of Environmental and Life Sciences, Poland. Wool samples for zinc determination were prepared by obtaining an ash, which was transferred to the flasks and supplemented with a suitable amount of deionized water. The content of zinc was measured on the AAS-3 atomic absorption spectrometer using a zinc lamp.

Wool fiber histological structure examination and a point analysis of elemental ion content were carried out using a scanning electron microscope (SEM) LEO 435 VP by Carl Zeiss SMT AG in the Laboratory of Electron Microscopy of the Faculty of Biology and Animal Breeding of the University of Life Sciences in Wrocław, Poland.

**Figure 1:**
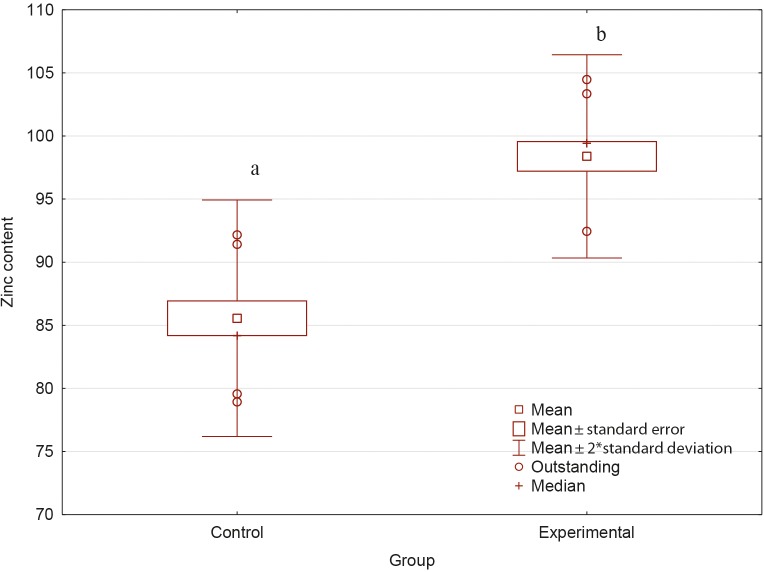
Zinc content in wool fibers from control and experimental groups (mg kg${}^{-1}$ DM). ${}^{a,b}$ shows differences significant at P≤0.05.

All statistical analysis was performed using STATISTICA v.10 (StatSoft, Tulsa, USA). The differences between the mean values were determined using the Student's t test for independent variables. Also the Spearmen correlation coefficients between the examined features was specified.

## 3 Results and discussion

Table 3 presents the mean values and standard deviations for the examined physicomechanical parameters of wool from the control and experimental groups. The mean length of sheep wool from the experimental group was 32 % higher compared to the control group. The average thickness of wool fibers from sheep from the experimental group was about 8 % higher in relation to the control group. The differences regarding the length and thickness of the examined samples were confirmed statistically (P<0.01). The values of mechanical properties of wool fiber, like breaking force and elongation, were also affected by supplementation. The breaking force was about 20 % higher (P<0.01), while the elongation value appeared to be about 4 % lower (P<0.05) in the experimental group compared to the control. The difference in the case of the tensile strength was not confirmed statistically; however, it tended to be slightly lower in the experimental group.

Zinc content in the analyzed wool fibers is presented in Fig. 1. The level of this element was significantly higher, by about 15 % (P<0.05) in the experimental group compared to the control (85.56 vs. 98.39 mg kg${}^{-1}$ DM).

**Table 4:** Correlations between the examined wool characteristics.

	Length	Thickness	Breaking	Elongation	Tensile	Zinc
	(cm)	(µm)	force	(%)	strength	content
			(N)		(N mm${}^{-2}$)	(mg kg${}^{-1}$ DM)
Length	1.000	0.544${}^{*}$	0.141	-0.117	-0.307	0.602${}^{*}$
Thickness		1.000	0.350	$-0.550^{*}$	$-0.506^{*}$	0.680${}^{*}$
Breaking force			1.000	0.002	0.627${}^{*}$	0.655${}^{*}$
Elongation				1.000	0.448${}^{*}$	-0.206
Tensile strength					1.000	0.065
Zinc content						1.000

${}^{*}$ – differences significant at P≤0.05.

**Table 5:** The content of some elemental ions on the wool surface (g 100 g${}^{-1}$).

Group	Element									
	C	O	S	Na	Ca	Mg	Al	Si	P	Zn
Control	58.63	29.74	5.73	0.01	0.62	0.16	0.31	0.50	0.73	0.01
Experimental	58.77	32.84	6.20	0.00	0,59	0.14	0.27	0.43	0.70	0.01

**Figure 2:**
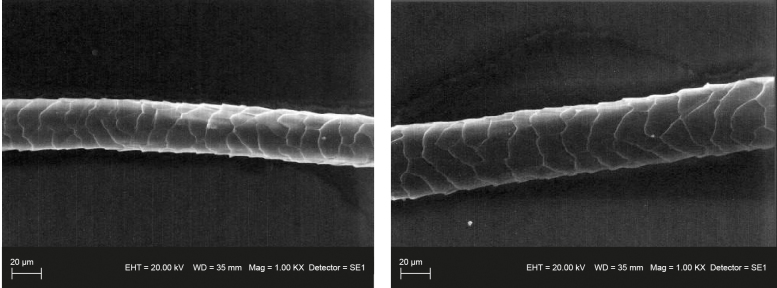
Histological structure of wool fiber from control and experimental groups.

The correlation coefficients between the analyzed wool fiber features are presented in Table 4. Statistically confirmed positive correlations were found between the value of length and thickness, length and zinc content, thickness and zinc content, breaking force and tensile strength and zinc content, and between the elongation and tensile strength. On the other hand, negative correlations were noted between the thickness of wool fibers and their breaking force and tensile strength.

An analysis of the percentage content of elemental ions on the surface of wool fibers was also performed. The results of this analysis are included in Table 5. There were no significant differences between the content of various elemental ions. In both groups, C, O and S, i.e., elements that build keratin, which is the basic hair protein, were the most abundant. The other elements, i.e., Na, Ca, Mg, Al, Si, P and Zn, occurred in much smaller amounts, below 1 g 100 g${}^{-1}$.

The histological structure of the wool fibers from both groups is shown in Fig. 2. The wool fibers of both groups were characterized by the correct construction of the cuticle. The scales were smoothly overlapping one another, both in the control and experimental group. The arrangement of cuticle scales was regular, ring-shaped, i.e., one scale surrounded the entire fiber, and their edges were smooth. The only difference visible in SEM images is the smaller diameter of fibers from the control group, which confirms the thickness measurements made using a projection microscope.

The last months of pregnancy and early lactation are the periods that not only negatively affect the quantity and quality of wool obtained from the ewes, but in the case of improper nutrition also the future wool production by their lambs (Corbett, 1979; Masters et al., 1993; Reis and Sahlu, 1994; Ferguson et al., 2011; Thompson et al., 2011). The length and diameter of wool fibers are the main parameters determining the price of wool. These are the features that are most influenced by proper nutrition (Reis and Sahlu, 1994; Sahoo and Soren, 2011). The main nutrients limiting the growth of wool are sulfuric amino acids (cystine, cysteine and methionine) and lysine, as well as zinc. Supplementation with sulfuric acids and zinc increases both the length and the diameter of the fibers (Page et al., 2017).

Our study demonstrated that wool of sheep supplemented with ZnMet bioplex was about 30 % longer and about 8 % thicker than wool of sheep fed without the addition. In the case of Merino sheep wool fibers, their thickness usually did not exceed 30 µm, and the average diameters is usually in the range of 16–26 µm (Holman and Malau-Aduli, 2012; Sharma and Pant, 2013), which is consistent with the results obtained in this study. White et al. (1994) showed a significant effect of zinc, in an inorganic form (${\mathrm{ZnSO}}_{4}$), on the growth of wool in the Merino breed rams in the case of zinc deficiency. It was also found in that study that zinc levels of only 4 mg kg${}^{-1}$ caused abnormal keratinization of fibers in the examined sheep and a significantly lower content of this element in their wool.

The physicomechanical properties of wool fibers are significant from a textile industry point of view, since they determine the usefulness and assignment of the fibers (Fatahi and Yazdi, 2010; Ragaišienė and Rusinavičiūte, 2012). The value of breaking force obtained is this study was higher in the experimental group compared to the control one, which was accompanied by higher thickness of wool fibers in the experimental group. This means that thicker fibers need a higher force to be broken, In turn, the elongation decreased with an increasing thickness of the fibers, which suggests that thicker fibers are less flexible. The value of tensile strength is one of the most important features determining wool fiber processing usefulness, and it represents the strength that should be applied to rupture the fiber. Higher values of this parameter demonstrate a higher resistance of the fibers. In this study, this parameter was not affected by the supplementation despite an increase in the values of thickness and breaking force in the experimental group. It can be thus concluded that the fibers after supplementation were stiffer and more resistant compared to the fibers obtained from sheep before supplementation.

Ryan et al. (2002) analyzed the bioavailability of zinc for adult Texel sheep. Using zinc in inorganic (${\mathrm{ZnSO}}_{4}$) and organic (bioplex) forms, they demonstrated that there is no difference between the amount of zinc from various sources incorporated into the wool. Concurrently, they proved that animals receiving zinc in organic form had significantly higher levels in their hooves and the quality of those hooves was better. Similar results regarding the lack of differences in the amount of zinc in wool depending on the source of its origin were obtained by Pal et al. (2010) and Page et al. (2017). Additionally, Page et al. (2017) showed no effect of zinc source on the increase in diameter and length of wool fiber in the growing rams. It was demonstrated in this study that the level of zinc in the wool fibers of supplemented sheep was about 15 % higher compared to nonsupplemented sheep.

However, many studies confirm better bioavailability of organic zinc forms, indicating its higher level in plasma, liver and other organs in animals supplemented with zinc chelates (Cao et al., 2000; Pal et al., 2010; Hassan et al., 2011).

## 4 Conclusions

Wool of sheep receiving the ZnMet bioplex addition was characterized by higher values of thickness, length and breaking force, with concurrently decreased elongation value, as well as a higher content of zinc in wool fibers. However, no significant differences were found in the content of zinc on the surface of the hair between the examined groups. It can therefore be concluded that the addition of the ZnMet bioplex positively affected the quality of wool obtained from sheep during pregnancy and early lactation with regards to physicomechanical parameters important from the processing point of view. Although an increase in wool fibers thickness was found, the values were still within the range reported in the literature for Merino sheep, and concurrently in the scope of the so-called fine fibers, most beneficial in the textile industry.

## Data Availability

The data sets are available upon request from the corresponding author.
